# The Differential Impact of Mystery in Nature on Attention: An Oculometric Study

**DOI:** 10.3389/fpsyg.2021.759616

**Published:** 2021-12-09

**Authors:** Alexandre Marois, Brooke Charbonneau, Andrew M. Szolosi, Jason M. Watson

**Affiliations:** ^1^École de Psychologie, Université Laval, Quebec City, QC, Canada; ^2^Thales Research and Technology Canada, Quebec City, QC, Canada; ^3^Department of Psychology, University of Colorado Denver, Denver, CO, United States; ^4^Department of Psychology, Montana State University, Bozeman, MT, United States; ^5^Department of Recreation, Sport Pedagogy, and Consumer Sciences, Ohio University, Athens, OH, United States

**Keywords:** nature, Attention Restoration Theory (ART), mystery, attention, eye tracking

## Abstract

Nature exposure can provide benefits on stress, health and cognitive performance. According to Attention Restoration Theory (ART), the positive impact of nature on cognition is mainly driven by fascination. Fascinating properties of nature such as water or a winding hiking trail may capture involuntary attention, allowing the directed form of attention to rest and to recover. This claim has been supported by studies relying on eye-tracking measures of attention deployment, comparing exposure to urban and nature settings. Yet, recent studies have shown that promoting higher engagement with a nature setting can improve restorative benefits, hence challenging ART’s view that voluntary attention is resting. Besides, recent evidence published by Szolosi et al. (2014) suggests that voluntary attention may be involved during exposure to high-mystery nature images which they showed as having greater potential for attention restoration. The current study explored how exposure to nature images of different scenic qualities in mystery (and restoration potential) could impact the engagement of attention. To do so, participants were shown nature images characterized by either low or high mystery properties (with allegedly low or high restoration potential, respectively) and were asked to evaluate their fascination and aesthetic levels. Concurrently, an eye tracker collected measures of pupil size, fixations and spontaneous blinks as indices of attentional engagement. Results showed that high-mystery nature images had higher engagement than low-mystery images as supported by the larger pupil dilations, the higher number of fixations and the reduced number of blinks and durations of fixations. Taken together, these results challenge ART’s view that directed attention is merely resting during exposure to restorative nature and offer new hypotheses on potential mechanisms underlying attention restoration.

## Introduction

With the omnipresence of technology and the high prevalence of urban or suburban life, nature exposure has significantly decreased over the last few decades. For instance, attendance to the main United States national parks has declined nearly 7% between 1993 and 2010 (Stevens et al., 2014). The COVID-19 pandemic also contributed to exacerbate this issue. In a recent survey conducted by Moore et al. (2020), it was reported that, during virus outbreak, time spent outside by Canadian children and youth reduced, whereas screen and social media time increased. Literature has, however, shown that nature exerts many positive impacts, including benefits for health (Bowler et al., 2010; Twohig-Bennett and Jones, 2018), affects (McMahan and Estes, 2015), stress (Hunter et al., 2019; Yin et al., 2020) and even cognition (Ohly et al., 2016; Stevenson et al., 2018). Considering the high demands known to incur from technology usage, multitasking and other daily life challenges, nature’s positive effect on cognition can be particularly useful to reduce fatigue or depletion. Such effects have been observed through lab and outdoor experiments, often relying on pre-post interventions to assess the potential recovery effects of nature on executive attention. Benefits have been observed on many cognitive activities including but not limited to creative problem solving (Atchley et al., 2012), working memory (Shin et al., 2011; Berman et al., 2012), inhibitory control of competing stimuli (Berman et al., 2008; Chung et al., 2018) and sustained attention (Berto, 2005; Pasanen et al., 2018).

According to Attention Restoration Theory (hereafter ART; Kaplan, 1995), nature’s benefits on attention originates from the characteristics nature inherently possesses. More precisely, ART posits that attention can be restored when the person-environment interaction has four factors. A person must first garner a sense of being physically or conceptually away from any distraction, worry or demanding situation from their daily life (being away). One must also be exposed to a context that is compatible with their purpose and inclinations (compatibility). Restorative environments must also capture visual attention with their fascinating properties (fascination) as well as be sufficiently rich and coherent such that attention is not only captured but also sustained (extent). Nature can help recover attention in response to these four properties; yet, fascination may play a particularly important role. Fascination is deemed effortless and automatic (James, 1892; Baumeister et al., 2000), typically involves attractive patterns difficult not to attend (Berto et al., 2010) and links to the tendency to process information under uncertainty (Kaplan and Kaplan, 1989; see also Mueller et al., 1972). At moderate levels—referred to as soft fascination—it is similar to James’ (1892) concept of involuntary attention (i.e., attention that requires no effort, triggered by a stimulus’ “direct exciting quality”), promoting a reduction in demands on executive-based attention (Kaplan, 1995, 2001; see also Pearson and Craig, 2014) and thus allowing directed attention to rest and recover as supported by improved performance on cognitive tasks observed at post-intervention assessments (e.g., Berman et al., 2008; Atchley et al., 2012; Sahlin et al., 2016). Fascination is driven by bottom-up processing and it differs from directed (or voluntary) attention, which rather refers to top-down effortful processes driven by inner intentions (Eimer et al., 1996). Both forms of attention have been associated with different origins and mechanisms of action in the brain (e.g., Näätänen et al., 1980; Mayer et al., 2004; Landau et al., 2007; Prinzmetal et al., 2009; but see Peelen et al., 2004). In ART, Kaplan proposed that nature can help recover the limited voluntary attention resources depleted by cognitively-demanding daily tasks because of its capacity to effortlessly capture involuntary attention (Herzog et al., 1997; Kaplan and Berman, 2010).

Consistent with ART, recent empirical work supports the notion that fascinating stimuli of nature can softly capture involuntary attention. For instance, Berto et al. (2010) fatigued participants before asking them to perform a Sustained Attention to Response Task (SART) where they needed to monitor sequences of built and nature environments of low and high fascination and respond on infrequent targets that appeared either on a cued position (valid trial) or at random (invalid trial). Their results showed that the cost/benefit difference in reaction time between valid and invalid trials was improved in the high fascination trials, but not in low fascination trials, for both images of nature and built environments. They interpreted this result as evidence that high fascinating stimuli reduced the need to suppress and inhibit distraction, hence facilitating the involuntary attention orienting toward the target. Hopman et al. (2020) relied on resting-state posterior alpha power changes to examine whether a multiday trip to nature could induce differences in introspection and internally-focused attention as compared with pre- and post-trip control testing. According to the authors, the lower resting state posterior alpha power changes they observed during the nature trip was consistent with ART’s view that nature exogenously captures involuntary attention, hence reducing possibilities for internal reflection (see also Ulrich et al., 1991; Chung et al., 2018; Olszewska-Guizzo et al., 2018; Scott et al., 2020).

A few studies have also directly investigated the role of attention through eye-tracking measures, including fixation, eye blinks and pupillary measures. Such metrics have been shown to provide a window to the deployment and engagement of visual attention and cognitive activity (e.g., Kahneman, 1973; Beatty, 1982; Culham et al., 1998; Van Orden et al., 2001; Irwin and Thomas, 2010; Chen et al., 2011; Shultz et al., 2011; Unsworth and Robison, 2017; Marois and Vachon, 2018). For instance, Berto et al. (2008) assessed the amount of engagement toward images of high-fascinating nature and low-fascinating built environments using fixations and saccadic movements measured by an eye-tracking system. Their results showed that low-fascination built (non-restorative) environments triggered more fixations than nature high-fascination images, suggesting inferior volition of attention during exposure to high-fascination nature. Valtchanov and Ellard (2015) also observed consistent results by showing a lower number of fixations, fixations of longer durations and a reduced amount of eye blinks when viewing nature scenes compared with urban scenes (see also Franěk et al., 2018). Moreover, Martínez-Soto et al. (2019) replicated the reduced number of fixations and their longer duration for nature restorative images compared with urban non-restorative images, but also showed higher pupil dilation for restorative nature images, which they ascribed to the superior affective preference for restorative nature environments. Overall, these results support ART’s interpretation that restorative environments elicit lower engagement of voluntary attention, particularly when comparing urban and nature scenes.

Yet, it has also been shown that attention restoration by nature may benefit from higher attentional engagement toward the restorative stimuli which contradicts the idea that attention would rest rather than being engaged toward restorative nature. For example, Duvall (2011) asked participants to take part in an outdoor-based intervention that consisted of taking at least three 30-min walks per week for 2 weeks. Participants were either assigned to a condition in which they had to develop a walking schedule with the researcher before the intervention (standard care group) or had to use awareness strategies regarding the environment while they were walking (engagement group). Results showed that subjects in the engagement group significantly improved their attentional functioning on a modified version of the Attentional Functioning Index (e.g., Cimprich, 1992). Pasanen et al. (2018) reached similar conclusions with people taking a single walk in an outdoor natural environment while being actively engaged toward the setting (e.g., looking around, finding one’s favorite place or searching for a place related to one’s situation in life). Indeed, participants who were asked to actively engage during their walk showed reduced commissions errors on the SART at post-test. These results strongly suggest that superior engagement and deployment of voluntary attention toward the restorative setting might be better for attention restoration (see also Lin et al., 2014; Browning et al., 2020). In fact, a recent eye-tracking study conducted by Stevenson et al. (2019) supported this conclusion. Specifically, the authors asked participants to either walk through a natural environment or urban environment while wearing a mobile eye tracker and performing the attention network task before and after the walk. Their results showed a faster and more stable pattern of results on the attention network task for participants that walked in nature, supportive of restoration of attention. Moreover, a higher fixation rate was observed for participants who walked in nature compared with those walking through a city. This finding supports the notion that voluntary attention might be active when one is being exposed to nature, which is in direct opposition with ART and with the other eye-tracking studies discussed above (cf. Berto et al., 2008; Valtchanov and Ellard, 2015; Franěk et al., 2018; Martínez-Soto et al., 2019).

One way to better understand the mechanisms underlying attention restoration by nature is to investigate the impacts of several nature settings characterized by different levels of restoration-promoting properties. This allows one to control for any potential specific nature-urban differences that might be difficult to disentangle in studies employing nature vs. urban comparisons (Scott et al., 2020). According to Szolosi et al. (2014), all nature is not equal, and more importantly, nature that specifically contains patterns of mystery may be particularly useful at opening opportunities for rest and mental state recovery given the known relationship between mystery and fascination (Herzog and Kropscott, 2004; Stamps, 2004). Here, mystery is defined as the characteristics of a setting that promote exploration, engaging and sustaining a person’s interest or fascination because of the sense that it can provide more than the information immediately available (see Kaplan and Kaplan, 1982, 1989; Stamps, 2004; Szolosi et al., 2014). It refers to settings that contain certain portions of the landscape that are obstructed or concealed (curvier patterns, richer in texture and poorer in color and luminance) such as a bend in a trail, a view hidden by foliage, or a stream that meanders out of sight (Hammitt, 1980; Gimblett et al., 1985). Given its ability to encourage one to venture, engage and discover more than there is—thus its close relation with fascination—mystery could offer benefits for attention restoration, especially given the aforementioned relation between engagement and restorative benefits (cf. Duvall, 2011; Lin et al., 2014; Pasanen et al., 2018; Browning et al., 2020). Therefore, manipulating mystery levels of nature images offer the possibility to control for restoration potential by relying only on nature images, putting aside any undesirable variability that would arise from urban settings.

Using a recognition memory task, Szolosi et al. (2014) showed that, with longer presentation duration at encoding (presumably permitting greater opportunity for deployment of voluntary attention), nature images perceived and rated high in mystery were significantly better recognized than those perceived low in mystery. Moreover, results on a self-reported perceived restoration scale supported that high-mystery images were considered more fascinating and restorative than low-mystery images. While investigating the impact of mystery and showing that it could lead to increased restorative benefits, Szolosi et al. (2014) also outlined an important aspect related to the role played by both voluntary and involuntary forms of attention during restoration. Given that involuntary processes are typically automatic and rapid while voluntary ones are controlled and contingent on greater time spent on task, manipulating image exposure time allowed them to simulate demand on attention and assess whether, at different durations, benefits could be observed. By observing that longer presentation durations entailed larger benefits, they showed memory performance was in fact related to the extent of directed/voluntary “effort” deployed with increased viewing time. Besides, the authors ran a mediation analysis and showed that the differences in benefits on recognition memory performance between both mystery sets were attributable to ratings in perceived fascination, but only for longer exposure durations (for 5 and 10 s, but not for 1 s). This outcome suggests that the positive effect of mystery, which in fact could be explained by differences in fascination levels, emerged only for longer periods wherein voluntary forms of attention could be deployed. Hence, from their perspective, nature endowed with higher restorative potential might be related to superior cognitive engagement and, therefore, higher recruitment of voluntary attention. To our knowledge, Szolosi et al. (2014)’s study represents the only investigation of the role of attention during restoration relying on two sets of nature images characterized by different restoration potential. However, Szolosi et al. (2014)’s evidence on the active role of attention remains indirect, meaning that the alleged superior engagement of attention during exposure to more restorative, high-mystery nature images was assessed through the differential impact of several exposure durations on recognition memory performance.

The goal of the current study was to follow-up on Szolosi et al. (2014) study by exploring how voluntary attention could be deployed while being exposed to nature images of different mystery levels, with presumably different potential for restoration. To reach this goal, participants were asked to actively look at 80 images (40 low- and 40-high mystery images which previously elicited restoration effects; see Szolosi et al., 2014) for 5,000 ms each and rate them on their fascinating and aesthetic properties. The objective of this task was to promote engagement of voluntary attention. While participants were deploying their attention toward the nature images, an eye tracker system collected measures of pupil size and eye fixations. Spontaneous eye blinks (see Espinosa et al., 2020) were also measured given their known relationship with cognitive functioning (e.g., Jongkees and Colzato, 2016; Eckstein et al., 2017). These measures were used to assess the extent to which participants were actively engaged toward each image of nature, thereby determining the impact of mystery on attention deployment given its positive relation with the number of fixations and pupil size, and its negative association with blink rate, blink duration and fixation duration (e.g., Kahneman, 1973; Beatty, 1982; Culham et al., 1998; Van Orden et al., 2001; Irwin and Thomas, 2010; Chen et al., 2011; Shultz et al., 2011; Unsworth and Robison, 2017; Marois and Vachon, 2018). Such a manipulation permitted attentional engagement with nature on a much shorter time scale than many ART intervention studies, skewing toward involuntary forms of attention in comparison with previous ART literature, but still with durations long enough to presumably engage voluntary attention controlled processes similar to Szolosi et al. (2014). Considering that participants were asked to actively (and voluntarily) look at the images and to rate them, if differences in attention engagement metrics should emerge, this would be attributable to a different level of voluntary attention engagement facilitated by the characteristics of each (high- or low-mystery) image.

In line with Szolosi et al. (2014), high mystery nature was expected to be perceived as more fascinating and rated with higher levels of likeability. We also anticipated increased attentional engagement as measured by the eye-tracking measures (i.e., increased pupillary dilations and fixation number, and reduced blinks and fixation duration) for high-mystery images compared with low-mystery images given Szolosi et al. (2014)’s demonstration that memory performance benefited from longer exposure durations, especially for high mystery images. Per ART’s perspective, the alternative hypothesis was that eye-tracking measures could rather support lower engagement (i.e., inferior pupillary dilations and fixation number, and increased blinks and fixation duration) for high-mystery images compared with low-mystery images given the notion that voluntary attention is expected to rest while one is exposed to restorative nature.

## Materials and Methods

### Participants

Fifty students from University of Colorado Denver (26 females, 22 males and two subjects self-identified as “other”) with a mean age of 21.2 years (*SD* = 3.6) volunteered to take part in this study in exchange for course credits. Participants were recruited from a subject pool developed at the University of Colorado Denver among students taking Psychology courses requiring them to participate in an Institutional Review Board-approved psychology experiment. The sampling method privileged was a non-probabilistic convenience method where all students interested could subscribe to take part in the experiment. Exclusion criteria comprised being more than 35 years of age. All reported having normal or corrected-to-normal hearing and vision.

### Apparatus and Material

**FIGURE 1:**
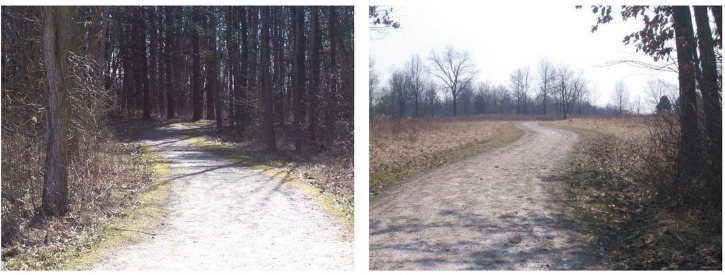
Example of the high-mystery (Left) and low-mystery (Right) images used in the current study (from Szolosi et al., 2014).

The experiment was conducted in a dimly lit room. A PC computer running an E-Prime 2.0 (Psychology Software Tools) program was used for presenting the instructions, controlling the presentation of the images and the questionnaires, and recording the responses. The images originated from Szolosi et al.’s (2014) stimulus set, each one being 640 pixels wide and 480 pixels high (see Figure 1). This image set contained 40 high- and 40 low-mystery nature settings (classification from normative data obtained^1^ by Szolosi et al., 2014) which previously led to high vs. low mystery differences in performance on a recognition task (Szolosi et al., 2014). Variations in the pupil size and eye coordinates were measured binocularly by using a Tobii TX300 eye tracker (Tobii Technologies) with a recording rate of 120 Hz. Each participant sat approximately 60 cm from the monitor on which the eye tracker was mounted. Although no chinrest was used, subjects were asked to keep their head movements to a minimum.

Participants performed a norming task in which they were asked to observe and rate nature images on their fascination and aesthetic properties. In each trial, following the presentation of a 48-pixel wide by 48-pixel high fixation cross for 2,000 ms, a nature image from either the low- or high-mystery category was presented on a 23-inch Dell monitor in a 1920 × 1080 resolution on a light gray background for 5,000 ms. Subsequently, two questionnaires were presented on the monitor in a random order. The *Shortened Perceived Restorativeness Scale* (Hartig et al., 1996) consisted of three, simultaneously displayed, questions originating from the fascination subscale of a longer scale designed by Korpela and Hartig (1996). This measure contained the following statements: “*This place has qualities that fascinate me*,” “*I would like to spend more time looking at the surroundings here*,” and “*My attention is drawn to many interesting things here*.” The fascination subscale of the *Shortened Perceived Restorativeness Scale* is focused on perceived restoration and is characterized by high internal consistency with Cronbach’s alpha values >0.80 (Hartig et al., 1996). Typically, higher scores on the PRS are assigned to soft fascinating/restorative nature while lower scores are generally associated with low restorative nature as well as environments typically referred to as hard fascinating (i.e., settings that drastically capture and consume attention resources such as urban scenery; see Korpela, 2013; Szolosi et al., 2014). For each image, participants indicated to what extent the statement described the experience they were having while looking at the image on a scale from 0 (Not at all) to 6 (Very much so). The *Likeability Scale* assessed the visual preference of an image. Participants were instructed to rate on a Likert scale from 1 (A strong dislike) to 7 (A strong preference) the extent to which they liked the image they had just seen. This measure was inspired by Kardan et al.’s (2015) work in which aesthetic preference, assessed by this single-item scale, was predicted by low-level visual features via a discriminant classification analysis.

### Procedure

After providing informed consent, the purpose of the experiment was briefly explained. The eye tracker was then calibrated. The experiment consisted of a norming task in which subjects were required to rate 80 images (40 low-mystery and 40 high-mystery) on their level of perceived fascination and likeability. Two blocks of trials, containing 20 images of each category, were performed in a counterbalanced order across subjects, interleaved with a self-paced break. Images were randomly presented within each block and both rating questionnaires were completed at each participant’s pace in a random order in each trial. Following the end of a trial, the next trial began automatically and immediately. Once the study was over, participants completed a brief sociodemographic survey, were debriefed, thanked, and credited for their participation. Subjects wore noise-reduction headphones to avoid any distraction from surrounding sounds.

### Analyses

#### Subjective Ratings

Global scores were calculated for each of the two measures and collapsed across all subjects to obtain one mean score per image. A *Perceived Fascination Mean Score* was calculated by averaging all three answers on the modified fascination subscale for each image for each participant, thus creating a mean score on a 7-point scale. An average of these mean scores was calculated across all subjects. The *Likeability Mean Score* also consisted of a mean score on a scale from 1 to 7 collapsed across all participants for each image. Scores were also averaged for each category (high vs. low mystery) to obtain one mean score for each type of trial per participant.

#### Eye-Tracking Preprocessing and Measures

Four participants were excluded as their data-recording rate was too low to compute valid eye measures (>50% missing data). Raw pupil data of the remaining participants were epoched from 200 ms before the presentation of an image to the end of the image presentation, 5,000 ms after its onset. An average of the pupil size for both eyes was computed to obtain only one measure. Data that were not rated as perfectly valid by the eye tracker software or that were recorded when a participant’s gaze was too distant from the image were withdrawn. Removed data and missing data points originating from malfunctions or eye blinks were linearly interpolated using MATLAB (MathWorks). Pupillary measures were then low-passed filtered using a cut-off frequency of 10 Hz, with a maximum attenuation in passband of 1 dB and a minimum in stopband of 40 dB (see Marois et al., 2018), resulting in an average of 6.3% of interpolated data among all participants.

Several eye-tracking measures were computed to assess the level of attentional engagement toward the to-be-rated images of nature. Absolute tonic pupil size (in mm) was first averaged for each subject within each mystery category, from 500 to 5,000 ms after the image onset. This 500--5,000-ms post-stimulus epoch avoided assessing attention engagement through pupillary measures that would be affected by the light reflex triggered by the presentation of the nature images^2^ (see, e.g., Labonté et al., 2019), while also potentially disentangling from transient mechanisms of involuntary attention capture typically occurring within the first 500 to 1,000 ms (Mathôt, 2018). Coordinates of the gaze on each image were also analyzed to assess the average number of fixations performed on each image (fixation/image) and the average duration (in ms) of those fixations for each subject on each type of trial. To be considered as a fixation, a participant’s gaze was required to be located in a given area of 0.5° for at least 60 ms. This fixation algorithm was based on the Dispersion-Threshold Identification fixation classification algorithm (see, e.g., Salvucci and Goldberg, 2000). Using Pedrotti et al.’s (2011) algorithm, spontaneous blink periods were identified as periods of eyelid closures, lasting between 50 and 500 ms, where no pupillary data could be measured. Once these blink periods were identified, a 60-ms period was added to each blink to consider the eyelid descent. Then, their frequency (in blink/image) and average duration (in ms) was measured in every trial and averaged for each subject within each mystery category.

#### Statistical Analyses

Ratings of fascination and likeability for both mystery sets were first compared from a within-subject perspective using paired-samples *t*-tests, but also compared at the image level between both mystery groups with two-sample *t*-tests. Measures of attentional engagement—that is tonic pupil size, eye blinks, and fixations—were analyzed with a within-subject design using paired-samples *t*-tests. To consider the potential influence of luminance, between-image analyses were also conducted for the pupillary measures. The critical α was fixed at 0.05 for each test. For each dependent variable, data points of ±3 *Z*-scores from the mean were identified as univariate outliers and were removed.

## Results

### Subjective Ratings

The Perceived Fascination Mean Score and the Likeability Mean Score were both compared across both types of mystery set to assess the restoration potential of the nature images. The 40 low-mystery images were rated an average of 2.36 points out of 6 (*SD* = 1.31) on the fascination scale, whereas the 40 high-mystery images reached a mean level of 2.82 points out of 6 (*SD* = 1.28). Although low at first glance, these fascination levels were similar to the mean levels on each image observed by Szolosi et al. (2014) and very strongly correlated (*r* = +0.90, *p* < 0.001). Moreover, the mean likeability level of low-mystery images was 3.60 out of 7 (*SD* = 1.15) and that of high-mystery images was 4.00 out of 7 (*SD* = 1.11)^3^. Paired-sampled *t*-tests showed that, as predicted, high-mystery images were considered significantly more fascinating, *t*(45) = 8.04, *p* < 0.001, Cohen’s *d* = 1.55, and were significantly more liked, *t*(45) = 10.35, *p* < 0.001, Cohen’s *d* = 1.17^4^. Results were also averaged within each block for every subject to test the reliability of the two measures. With Cronbach’s alpha of 0.97 and 0.95, the fascination and likeability scales, respectively, demonstrated very strong reliability. Taken together, these results replicated Szolosi et al.’s (2014) findings showing that high mystery was related to higher ratings of perceived fascination and aesthetic preference while being consistent with the view that mystery is a predictor of preference (cf. Herzog and Kropscott, 2004; Stamps, 2004; Herzog and Bryce, 2007).

### Eye-Tracking Measures of Attentional Engagement

#### Pupillometry

**FIGURE 2:**
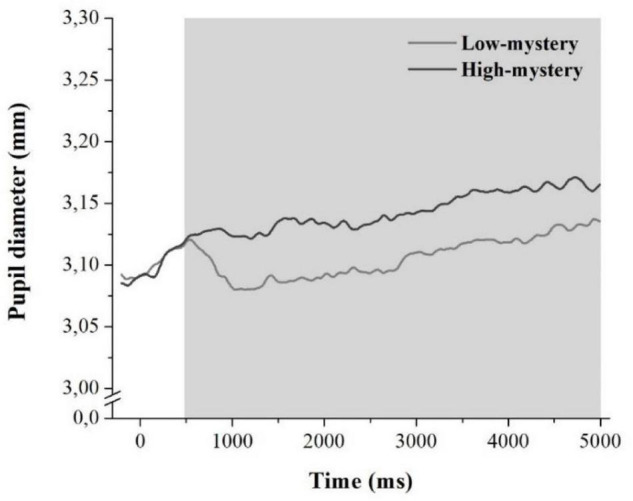
Variation of the mean pupil diameter (in mm) as a function of time (in ms) for both low-mystery (solid gray line) and high-mystery trials (solid black line) averaged across all participants. Time 0 represents the onset of the image that is displayed for 5,000 ms. The gray area represents the time-window during which the mean tonic pupil diameter was computed.

Figure 2 displays the variation in the pupil size averaged for both low- and high-mystery trials for all participants. The mean tonic pupil diameter observed in the 500–5,000-ms time-window was 3.11 mm (*SD* = 0.44) in low-mystery trials and 3.14 mm (*SD* = 0.44) in high-mystery trials. The paired-samples *t*-test showed that the averaged tonic pupil diameter was larger when subjects looked at high-mystery images than when they looked at low-mystery images, *t*(45) = 6.71, *p* < 0.001, Cohen’s *d* = 0.76.

It could, however, be argued that potential visual differences in the images used for both low- and high-mystery sets, especially differences in brightness, may explain why the tonic pupil size differed between these two categories. Higher luminance levels are indeed known to produce constriction in the pupil size, as opposed to darker images that are known to cause pupil enlargement (see, e.g., Winn et al., 1994; Mathôt et al., 2014; Peysakhovich et al., 2015). To test this hypothesis, we analyzed the brightness level of each image following Berman et al.’s (2014) method. As expected and consistent with previous literature on mystery (cf. Gimblett et al., 1985; Herzog and Miller, 1998; Schertz et al., 2018), low-mystery images (*M* = 0.57, *SD* = 0.03) were significantly brighter than high-mystery images (*M* = 0.53, *SD* = 0.04), *t*(78) = −3.96, *p* < 0.001, Cohen’s *d* = −1.13. Despite the tonic pupil diameter being computed later in the signal, when the light reflex is hypothesized to be completed (Lowenstein and Loewenfeld, 1952a,b; Beatty and Lucero-Wagoner, 2000; Mathôt et al., 2018; Labonté et al., 2019), one could contend that the increased pupil size observed in high-mystery trials still originated from the luminance features of the images, especially because high-mystery images are darker, which elicits pupil enlargement.

**FIGURE 3:**
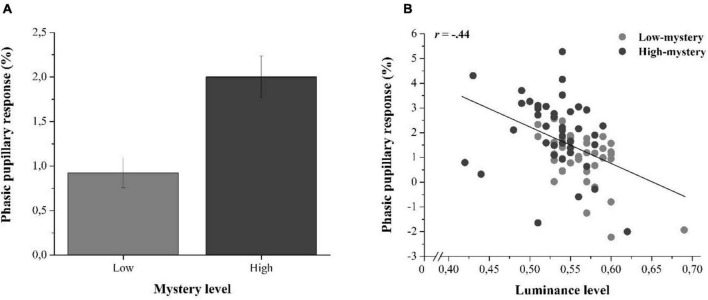
**(A)** Averaged phasic pupillary responses (in %) elicited by the nature images presented in low- and high-mystery trials. Error bars represent the standard error of the mean. **(B)** Scatter plot illustrating the relationship between the luminance level and the phasic pupillary response (in %) as a function of the type of trial.

To address this possibility, we computed image-elicited phasic pupillary responses in each trial and averaged them across all participants to obtain one mean measure of phasic pupil response per image. These phasic pupillary responses were measured by quantifying the mean amplitude of the pupil size over the 500–5,000-ms time-window from which a 200-ms-prestimulus baseline value (i.e., before the onset of the image, during fixation) was subtracted. This difference in pupil amplitude was then divided by the baseline value to obtain a percentage of variation in pupil size (%; see, e.g., Marois et al., 2018). An image-oriented independent *t*-test showed that the pupillary response triggered by high-mystery images (*M* = 2.00%, *SD* = 1.50) was significantly larger than that elicited by the low-mystery images (*M* = 0.93%, *SD* = 1.06), *t*(78) = 3.70, *p* < 0.001, Cohen’s *d* = 0.82 (see Figure 3A). A Pearson correlation analysis then showed that these pupillary responses triggered by the presentation of the nature images were significantly correlated with measures of brightness, *r*(78) = −0.44, *p* < 0.001 (see Figure 3B), supporting the relationship between brightness and change in pupil size.

To test whether the impact of mystery category was still significant without the influence of luminance, we then performed an Analysis of Covariance (ANCOVA) on the phasic pupillary responses with the mystery category as the main factor and the luminance level as the covariate. This analysis revealed that although the impact of luminance on the phasic pupillary response was significant, *F*(1,77) = 9.99, *p* = 0.002, $\eta_{\text{p}}^{2}$ = 0.12, there was still a significant main effect of mystery category when statistically controlling for luminance, *F*(1,77) = 5.15, *p* = 0.026, $\eta_{\text{p}}^{2}$ = 0.06, with a semi-partial correlation of s*r* = +0.46 (*p* < 0.001). Hence, this analysis supported the idea that high-mystery images with greater attention restoration potential elicited larger pupillary responses that were not strictly due to their reduced brightness relative to low-mystery images.

#### Fixations and Blinks Analyses

Measures of fixations and eye blinks were collected using the gaze coordinates and the missing data points in pupil size. They were used as alternative measures of attention engagement because of their robustness to visual characteristics of the images such as luminance and color (Helland et al., 2007; Açik et al., 2009). One subject was excluded from the fixation analysis and two others from the blink analysis as their data were rated as outliers. Table 1 shows the means and standard deviations for the average number of fixations per image, the average number of blinks per image, the average duration of the fixations, and the average duration of the blinks for each type of image (either low- or high-mystery).

**TABLE 1:** Mean, standard deviation (in parentheses) and results of the paired-sample *t*-tests for the four measures of blink and fixation calculated for each type of mystery category.

Measure	Mystery category		*t*	Cohen’s *d*
	Low	High		
Average number of fixations (fixation/image)	15.45 (4.47)	15.93 (4.36)	3.79***	0.57
Average number of blinks (blink/image)	1.52 (0.94)	1.40 (0.85)	−2.82**	−0.43
Average fixation duration (ms)	222.00 (83.88)	216.80 (76.64)	−2.17*	−0.33
Average blink duration (ms)	215.01 (42.40)	212.01 (40.64)	−1.23	−0.18

*p < 0.05, **p < 0.01, ***p < 0.001.

Paired-sample *t*-tests showed that the average number of fixations per image was significantly greater for high-mystery images than for low-mystery images, *t*(44) = 3.79, *p* < 0.001, Cohen’s *d* = 0.57. The opposite pattern was observed for the average number of blinks per image as significantly more blinks were performed in low-mystery trials in comparison with high-mystery trials, *t*(43) = −2.82, *p* = 0.007, Cohen’s *d* = −0.43. The average fixation duration was also significantly longer for low-mystery images than for high-mystery images, *t*(44) = −2.17, *p* = 0.036, Cohen’s *d* = −0.33. Finally, no difference was observed in the average blink duration between low- and high-mystery images, *t*(43) = −1.23, *p* = 0.226, Cohen’s *d* = −0.18. These analyses showed that high-mystery images elicited more fixations, less blinks, and fixations of shorter duration compared with low-mystery images. This is consistent with the larger increase in pupil size observed for high-mystery images and with the hypothesis that nature images with allegedly greater restorative potential (i.e., nature endowed with more mystery, eliciting higher engagement; Kaplan and Kaplan, 1982, 1989; Szolosi et al., 2014) promote more attention volition.

## Discussion

The current study provides one of the first direct explorations of the deployment of attention while viewing nature images of different mystery levels, i.e., with allegedly different potential for restoration (cf. Szolosi et al., 2014). To reach this goal, we collected eye-tracking measures (i.e., pupil size, fixations, and blinks) to investigate whether participants’ attention would be more engaged and active while viewing high-mystery images compared with low-mystery images using the same set of pictures as Szolosi et al. (2014). Participants were also asked to provide ratings of likeability and fascination on a subscale of the *Shortened Perceived Restorativeness Scale*. Results first replicated the higher ratings of fascination observed by Szolosi et al. (2014) for high-mystery images, as well as extended these findings to demonstrate high-mystery images were also more liked. More importantly, eye-tracking measures showed that attention was more active and engaged when participants were looking at images with more mysterious features, providing evidence toward a greater deployment of voluntary attention for these settings as participants were asked to actively look at the images for 5 s before rating them on their fascination and aesthetic properties. Indeed, for high-mystery trials, pupil size—even when controlling statistically for luminance variability—was larger, fixations were more frequent and of shorter duration, and eye blinks were less frequent. Given the relationship previously demonstrated between attention involvement, pupil size, fixations, and blinks (Kahneman, 1973; Beatty, 1982; Culham et al., 1998; Van Orden et al., 2001; Irwin and Thomas, 2010; Chen et al., 2011; Shultz et al., 2011; Unsworth and Robison, 2017; Marois and Vachon, 2018), these results support the view that raising the mystery level in nature increases one’s attentional engagement which in turn potentially augments the restorative potential of nature (cf. Duvall, 2011; Lin et al., 2014; Pasanen et al., 2018; Browning et al., 2020).

The fact the Szolosi et al. (2014) observed better memory for the more mysterious nature images is consistent with our demonstration of higher engagement for those same images. In fact, literature has shown that improved memory performance at recall was related to higher engagement and more optimal deployment of attention at encoding as supported by larger pupil sizes, increased visual exploration and less eye blinks (Shultz et al., 2011; Irwin, 2014; Kucewicz et al., 2018; Damiano and Walther, 2019; Meghanathan et al., 2019; Miller et al., 2019). These results also provide evidence in favor of the potentially active role of voluntary attention during restoration reported by Szolosi et al. (2014). In their study, longer viewing durations incurred higher demands on voluntary attention processes that are controlled and sustained as opposed to involuntary attention mechanisms known to be transient, rapid and automatic. This increased exposure duration led to superior benefits for memory performance (Posner et al., 1980, 1982; Wright and Richard, 2000). Here, we show that the same set of high-mystery images indeed leads to a higher attention engagement that can potentially be ascribed to voluntary mechanisms.

While no actual measure of restoration was collected, our findings are also consistent with studies in which a superior restoration experience was related to higher engagement toward nature (e.g., Duvall, 2011; Lin et al., 2014; Szolosi et al., 2014; Pasanen et al., 2018; Browning et al., 2020). In these studies, the positive impact of nature exposure on cognitive performance was amplified when participants performed tasks that promoted their engagement toward the nature setting (e.g., actual vs. virtual exposure, enhanced awareness toward the nature setting or stimuli, or longer viewing time). The fact that these studies showed optimized restoration with higher engagement contradicts ART’s view that voluntary attention is resting when one is exposed to nature, and thus puts our results into context. As shown by Szolosi et al. (2014) and supported by other literature showing a positive relationship between fascination and mystery (cf. Kaplan and Kaplan, 1982, 1989; Herzog and Kropscott, 2004; Stamps, 2004), higher levels of mystery can engage one’s attention toward nature scenes and in turn improve the restorative experience. Because we observed higher measures of attentional engagement, this suggests that settings characterized by greater restoration potential tend to engage one’s voluntary attention.

The observation of higher voluntary attentional involvement for the settings containing more restoration potential (i.e., higher levels of mystery and higher fascination ratings) seem at first glance to contradict most of the eye-tracking studies focused on the deployment of visual attention during interaction with nature (Berto et al., 2008; Valtchanov and Ellard, 2015; Franěk et al., 2018; Martínez-Soto et al., 2019). In these studies, participants were typically exposed to nature and urban settings either in real or virtual form (i.e., using images), and oculometric measures were collected to infer their attention deployment toward the settings. Supported by these metrics, the authors observed that urban environments were generally related to higher involvement of voluntary attention (e.g., more fixations and larger pupil dilation) when compared to nature environments. Consistent with ART, they interpreted this pattern of results as evidence that voluntary attention is resting during its restoration because nature—which led to inferior attention involvement—did contribute to attention restoration, as opposed with urban environments.

Yet, to fully explore the mechanisms of attention restoration, one must also compare nature environments of different restoration potential. Our investigation thus focused on comparing two sets of nature images of different restoration potential and contradicted these studies. An explanation for such an opposite view can originate from the type of fascination that is triggered by nature and urban images. According to Kaplan and Berman (2010), urban environments tend to trigger hard rather than soft fascination, which contains bottom-up stimulation that automatically captures attention and requires directed attention to resist. This explains why urban environments may be less restorative than nature settings (Berman et al., 2008). Nature settings can trigger soft fascination and the attraction they typically elicit does not incur resistance nor inhibition that might require executive-based attention (Kaplan, 1995, 2001; Pearson and Craig, 2014). Exposure to (softly fascinating) nature setting thus entails lower engagement than urban environments because no effort is required to inhibit the “competing” attraction driven by nature. Yet, one could also be exposed to nature settings that are considered less fascinating, with stimuli that might be less preferred and cause reduced attention deployment. Such would be the case for low mystery nature settings, as opposed to high mystery nature settings stronger in the fascination they elicit. Considering both our results and those raised by the other eye-tracking studies, a pattern can emerge.

**FIGURE 4:**
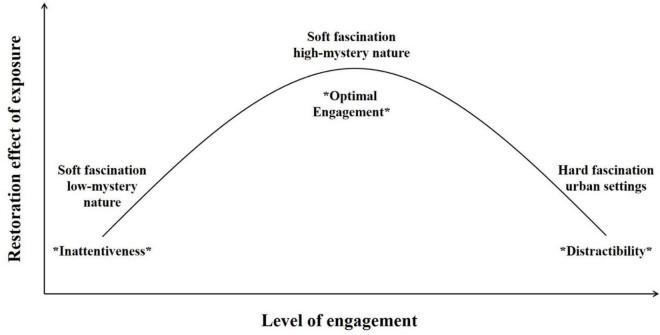
Hypothesized relationship between engagement and attention restoration.

Here, we propose that the relationship between attentional engagement of the environment and restoration potential may be characterized by a quadratic trend, in the form of an inverted-U shaped relationship. At the lower level of the engagement continuum, suboptimal attention restoration may be possible with nature settings that are less captivating (e.g., low mystery settings) where one would be less involved toward the setting, in a state that might approach inattentiveness. At moderate levels of engagement, optimal attention restoration might be related to soft fascination with nature settings sufficiently captivating (e.g., high mystery images) to attract one’s attention without consuming it and drawing from executive attention. On the highest end of the continuum, urban settings characterized by hard fascinating stimuli may engage one’s attention even more than high-mystery nature images while also consuming it, thereby reducing their potential for restoration. Such a situation could be described as a state of distractibility, where one’s mental resources would be too involved toward the urban stimuli to allow voluntary attention to restore. Figure 4 depicts such a curvilinear relationship between attention engagement and restoration potential according to the type of exposure. Although this hypothesis admittedly goes slightly beyond our results, it does reconcile the observation of higher engagement toward (non-restorative) urban settings reported by previous eye-tracking studies with our observation of higher engagement toward (more restorative) high-mystery nature images, compared with (less restorative) low-mystery nature images. However, further investigation is needed to better understand the differences in attention involvement toward these three types of settings and to fully explore the relationship between engagement and restoration. Different types of urban scenes should also be compared, manipulating the level of fascination and engagement they elicit to investigate this potential moderating effect on attention restoration as well (see, e.g., the urban control environments of Hopman et al., 2020; Scott et al., 2020, which somewhat counterintuitively elicited *lower* levels of engagement than nature environments such as a sandy riverbank in the desert, perhaps because participants in the urban condition were sitting outside on a familiar college campus looking at a concrete wall).

Per our results, voluntary attention would be engaged in the attention restoration process. Yet, an alternative hypothesis remains. Although the superior cognitive engagement observed for high-mystery images for the 500–5,000 ms time-window could be specifically ascribed to mechanisms of voluntary attention typically observed on longer and sustained periods (e.g., Wright and Richard, 2000), one could still argue that it rather represents mechanisms of involuntary attention in a sense similar to what is proposed by ART. Recall that, per ART’s viewpoint, involuntary attention would be captured by the fascinating stimuli of nature, then reducing further voluntary attention demands on the system, allowing at the same time restoration of executive attention. Although the effects observed occurred on a longer period and hardly represent involuntary mechanisms (which are normally automatic and fast-acting; see Posner et al., 1980, 1982), disentangling the voluntary and involuntary mechanisms of attention deployed while participants looked at the nature images may be difficult. In light of our results, it seems, however, more likely that voluntary attention processes were engaged, although this does not exclude any previous/automatic involvement of involuntary attention, especially given the previous demonstration of superior involuntary attention engagement when interacting with restorative nature scenes (cf. Berto et al., 2010; Olszewska-Guizzo et al., 2018; Hopman et al., 2020).

However, one may wonder how increased attention engagement toward the nature setting might contribute to the restoration of cognitive (and allegedly voluntary attention) resources (Kaplan, 1995; Berman et al., 2008; Kaplan and Berman, 2010). As suggested by Szolosi et al. (2014), those attentional benefits might originate from a balance between involuntary and voluntary attentional networks. Specifically, more mysterious nature and their physical properties likely seem to evoke a sense of soft fascination, and this type of fascination can engage a person’s attention fairly automatically. In turn, instead of using cognitive resources to strictly inhibit (automatically and involuntarily) responding to stimuli that may demand high involvement of executive attention (i.e., for urban scenery, similar to the word dimension in a Stroop color naming task; Stroop, 1935, where individuals must overcome oppositional logic to correctly name ink colors rather than to incorrectly read words), we propose the mystery and soft fascination inherent in nature may facilitate the engagement of a person’s voluntary attention, hence reducing cognitive demands and providing an explanation to the cognitive benefits typically ascribed to restoration by nature. This would be in line with the bulk of the visual-spatial literature showing that exogenous (involuntary) cueing can in turn facilitate sustained (voluntary) processing of visual information (e.g., Fernandez-Duque and Posner, 1997; MacLean et al., 2009; Dugué et al., 2020; Nguyen et al., 2020). Critically, this view would also reconcile both evidence that involuntary attention is automatically and effortlessly captured (e.g., Berto et al., 2010; Olszewska-Guizzo et al., 2018; Hopman et al., 2020) with that related to the benefits of superior attention engagement toward the nature setting (Duvall, 2011; Lin et al., 2014; Szolosi et al., 2014; Pasanen et al., 2018; Stevenson et al., 2018). Even so, future work should specifically investigate this switch in the form of attention that is engaged (from automatic involuntary to controlled voluntary) to better understand how both forms of attention may be involved during exposure to restorative nature.

### Study Limitations and Avenues for Future Research

This experiment possesses some limits that may be addressed in future research. First and foremost, the important differences in luminance between high and low mystery images represent a limitation in the differences observed in pupil size between both types of images. Mystery is characterized by curvier pathways, partial concealment and shadows (Gimblett et al., 1985; Herzog and Miller, 1998; Schertz et al., 2018). Hence, this type of image is intrinsically characterized by such visual features, thereby limiting the possibility to experimentally control this confounding variable. Controlling statistically for the variance explained by luminance still led to a significant effect of type of image, but this effect was also low ($\eta_{\text{p}}^{2}$ = 0.06). One solution to this was to rely on other eye-tracking measures known to be impervious to luminance variations (Helland et al., 2007; Açik et al., 2009). Again, these measures led to significant differences between high and low mystery images with effect sizes varying from low to moderate (Cohen’s *d*s = −0.33, −0.43, and 0.57). One explanation for these relatively low effect sizes might be the important similarities between images of the low and high mystery nature sets (see the Supplementary Material of Szolosi et al., 2014; see also the Supplementary Material for a comparison of the low-level visual properties of the two different mystery sets following the method employed by Berman et al., 2014). Typically, studies interested in attention restoration rely on images of nature vs. urban settings; consequently, effect sizes can be exacerbated by other components not necessarily related to the manipulation *per se*, but also to the visual characteristics inherent to nature and urban settings (Berman et al., 2014; Scott et al., 2020). Here, relying on two sets of nature images similar in appearance might have reduced the amplitude of the mystery effect, though significant results were still observed. Future studies should attempt to reproduce the current pattern of results with other sets of high and low mystery nature images that are similar (to replicate our findings) but also different from one another (to extend the current findings, perhaps even including a variety of urban images as well).

Second, one could argue that a 5-s exposure in a lab might not be long enough or ecologically-valid enough to detect actual attention involvement toward the nature settings. Such duration has, however, been shown to be sufficiently important for nature images to produce benefits on attention (cf., the recognition memory results of Szolosi et al., 2014). Concerning the potential lack of authenticity, previous studies have successfully used controlled laboratory experiments, performed by volunteer college students, to assess nature’s positive impact on cognitive measures (e.g., Berto, 2005; Berman et al., 2008). Moreover, Hartig et al. (1997) have shown that perceived fascination measures were consistent whether participants interacted with actual or pictured natural settings (see also Stamps, 1990, for a meta-analysis regarding preference consistency between *in situ* and photographed environments; but see Stevenson et al., 2018). Relying on a lab study also permitted a test of boundary conditions of an “involuntary attention alone” hypothesis, allowing an assessment of rapid automatic/involuntary mechanisms as well as longer controlled/voluntary effects, where the latter have been partially demonstrated in real-life conditions (Hopman et al., 2020; Scott et al., 2020). Additionally, and despite previous criticisms reported for the PRS ability to objectively represent fascination and to distinguish between soft and hard fascination (see, e.g., Basu et al., 2018; Neilson et al., 2019), the relatively low levels of fascination reported by the participants for each type of image (*M* = 2.36 for low-mystery images, *M* = 2.82 for high-mystery images) could challenge the idea that these images are endowed with restoration potential. While those levels are strongly correlated (*r* = +0.90) to the ratings of Szolosi et al. (2014) and relatively similar (*M* = 2.59 for low-mystery images, *M* = 3.11 for high-mystery images, with a mean difference of 0.26 points), we acknowledge that those levels seem different from those typically observed in ART literature (e.g., similar to the low fascination scores reported for non-restorative urban images in Berto, 2005). The relationship between restoration potential and levels of attention engagement toward an environment/activity can, however, be seen as a continuum, starting from very low to high fascination where different levels lead to different forms and levels of attention engagement/consumption (Szolosi et al., 2014; Basu et al., 2018). Here, as explained earlier, the images that were used were highly similar to one another and may represent a specific part of this continuum with potentially lower—but still present—potential for restoration. Moreover, despite these low differences in fascination reports, we did empirically observe differences in attention engagement, and even in cognitive benefits in previous studies (cf. Szolosi et al., 2014), hence supporting functional differences in the effects generated by these two sets of images. Nevertheless, future studies could attempt to replicate our results by using varying exposure durations with the images to determine whether measures of attention might change with greater or lesser time-on-task, and presumably, engagement with the nature images. It may also be worthwhile for future work to assess whether our findings would generalize with environments, whether actual or pictured, that would differ more and thus cover a higher spectrum in terms of mystery, fascination, attentional engagement, and restoration potential.

Third, the present experimental design differed from classic ART studies where, following a fatigue-inducing task, participants are exposed to either urban or nature images to examine the restoration effect on a given cognitive task. Even so, other studies have relied only on nature and manipulated aspects such as the level of engagement (Duvall, 2011; Lin et al., 2014; Pasanen et al., 2018) or characteristics of the settings (Szolosi et al., 2014). Besides, Scott et al. (2020) pointed out that comparisons with urban settings also add potential confounds that may be difficult to disentangle, especially if one needs to investigate a specific component related to nature’s restoration potential. Regarding the absence of resource-depleting task before the presentation of the nature images, even if mental fatigue is considered highly prevalent among college students (Kaplan, 1995; Tennessen and Cimprich, 1995; Versaevel, 2014), our goal was not necessarily to assess the restoration potential of the nature images, but rather to examine how attention would be deployed. As such, the absence of fatigue among the participants might not impact our comparisons between low and high mystery nature settings. However, future work could extend the current investigation to include urban images perceived either low or high on their mystery properties as well as depleting participants’ attentional resources before being exposed to the nature or urban images to assess whether directed attention would be engaged differently. A more classic intervention approach using these sets of images could also be privileged to replicate the restorative benefits of mystery and further support the benefits of engagement for attention restoration by this type of nature.

Finally, the fact that the current pattern of results demonstrates activation of directed attention could also be challenged by the validity of ART itself. While ART represents one of the main theoretical approaches used in the literature to explain the empirical benefits observed following exposure to restorative nature, it is also highly criticized. Joye and Dewitte (2018) provide a comprehensive criticism of the empirical and conceptual limitations of ART. In fact, while reporting relevant literature to support their claim, they suggest that: (a) supports of actual cognitive restoration—and specifically of directed attention—still remains tenuous; (b) the concept of soft fascination is too vague and lacks clear operationalization; (c) the notion of effortless attention recovery by nature settings is elusive; and (d) the idea that it relies on evolutionary and adaptive mechanisms is insufficiently supported. At first glance, our results may provide some answers to Joye and Dewitte’s critique. Indeed, our results of superior attention engagement toward high-mystery (and allegedly restorative) nature support the idea that ART’s notion of “effortless” attention recovery may be incorrect. What is more, the differences in attention engagement, likeability levels and in low-level visual properties—including luminance, but also curvilinear lines, color saturation and entropy (see the Supplementary Material)— can also help to better operationalize the concept of fascination. Yet, our interpretation largely relies on the distinction between directed (voluntary) and automatic (involuntary) attention during exposure, a phenomenon challenged by Joye and Dewitte. Regardless, we still demonstrated differences in attention engagement while participants were exposed to two sets of mysterious nature being characterized by different visual properties and rated at distinct levels on their fascination and likeability levels. From an empirical point of view, these results still shed light on the deployment of (visual) attention that can ensue from distinct levels of fascination and interest toward a nature visual scene. In this light, the current paper adds to a growing number of recent studies demonstrating greater psychophysiological responses when interacting with nature, suggesting greater involvement of attentional networks and emphasizing behavioral responses to nature as a stimulus, rather than focusing on restoration outcomes in response to nature *per se* (see Hopman et al., 2020; LoTemplio et al., 2020; Scott et al., 2020).

## Conclusion

Within classical literature, authors have lauded nature for its ability to provide a venue that can facilitate cognitive clarity (Emerson, 1836; Thoreau, 1854). That is, nature seems to offer many people a place that can engage their attention effortlessly, while at the same time allowing them to maintain some attentional volition. Findings from our study align well with this notion. Interactions with certain natural settings appear to engage a form of attention that resonates with both bottom-up (involuntary) and top-down (voluntary) cognitive processes. Hence, interacting with nature may not permit directed attention to rest, but rather engages it in a way that resonates with bottom-up visual properties of the environment. Such resonance may serve to recalibrate flexible attention’s relationship with the environment, moving away from top-down inhibition to sources of interference that may be cognitively depleting and effortful toward the capture of attention by fascinating stimuli, thereby facilitating its restoration. The culmination of this work could eventually lead to the development of cognition-supportive systems or interventions that might be used by workers, students, or simply people who seek attention recovery following—or preceding—any period of cognitive fatigue. The stress and the cognitive challenges (e.g., interruptions, distractions, rapid and complex problem solving, dual tasking, information retention, and long periods of vigilance) that students (e.g., Misra and McKean, 2000; Robotham and Julian, 2006; Versaevel, 2014; Guerra-Carrillo et al., 2017) and workers from an office (e.g., Young and Berry, 1979; Jett and George, 2003; Crompton, 2011; Mak and Lui, 2012) or high-risk domains (e.g., Dehais et al., 2014; Grier, 2015; Hodgetts et al., 2017; Marois et al., 2019) confront might be mitigated by using these interventions.

In conclusion, future work should consider the extent to which interacting actively with high-mystery nature scenes translates into secondary benefits on cognitive tests but especially measures of executive attention. Further investigation of the proposed attentional resonance between top-down and bottom-up forms of processing is also needed to assess how perception, deployment of attention, memory, and even internal thoughts (mind wandering) can be impacted by mystery, fascination or other image properties. For example, these studies could provide converging evidence on whether cognitive engagement with high-mystery, and thus more fascinating, nature settings may impact mind wandering thoughts compared to low-mystery settings. This could provide additional evidence of the active engagement of directed attention during restoration by nature.

## Data Availability Statement

The raw data supporting the conclusions of this article will be made available by the authors, without undue reservation.

## Ethics Statement

The studies involving human participants were reviewed and approved by Colorado Multiple Institutional Review Board, University of Colorado Denver. The patients/participants provided their written informed consent to participate in this study.

## Author Contributions

AM and JW developed the study concept and experimental design. AM and BC contributed to the acquisition and analysis of the data under the supervision of JW. All authors took part in the interpretation of the data. AM, JW, and BC drafted the manuscript and key revisions were provided by AS. All authors approved the final version of the manuscript for submission.

## Conflict of Interest

AM was employed by the company Thales Research and Technology Canada. The remaining authors declare that the research was conducted in the absence of any commercial or financial relationships that could be construed as a potential conflict of interest.

## Publisher’s Note

All claims expressed in this article are solely those of the authors and do not necessarily represent those of their affiliated organizations, or those of the publisher, the editors and the reviewers. Any product that may be evaluated in this article, or claim that may be made by its manufacturer, is not guaranteed or endorsed by the publisher.

## Footnotes

1 The complete image set, as well as the Szolosi et al. (2014) normative data for mystery and fascination ratings, can be found online at: http://www.frontiersin.org/journal/10.3389/fpsyg.2014.01360/abstract.

2 According to Beatty and Lucero-Wagoner (2000), the peak of the pupillary light reflex occurs within a timeframe of 500 and 1,000 ms following the change in luminance (see also Lowenstein and Loewenfeld, 1952a,b; Mathôt et al., 2018). The latency of this reflex is however, dependent on the intensity of the luminance change, but also on the duration of the light-changing stimulus (e.g., Wang et al., 2018). Because the presentation duration of the nature images was the same regardless of the type of trial, the same time-window for the light reflex was used for low- and high-mystery images. More specifically, this 500–5,000-ms time-window was chosen following a visual inspection of the average pupillometric signal where we identified the peak of that reflex as occurring around 500 ms post-stimulus (see Figure 2).

3 The fact that fascination levels can be considered relatively low but that the levels of likeability can be considered moderate (i.e., a bit superior to mid-scale) supports that the nature images most likely did not elicit hard fascination. This type of fascination is mainly associated with attention-consuming urban images and inferior reports of preference (Valtchanov and Ellard, 2015).

4 Note that measures of likeability and fascination were also averaged among all participants for each image to conduct an item-based analysis. Two-sample *t*-tests supported that the difference between low and high mystery images was statistically significant for the likeability score [low-mystery images: *M* = 3.60, *SD* = 0.35; high-mystery images: *M* = 4.00, *SD* = 0.42; *t*(78) = 4.66, *p* < 0.001, Cohen’s *d* = 1.04] and for the fascination score [low-mystery images: *M* = 2.36, *SD* = 0.36; high-mystery images: *M* = 2.82, *SD* = 0.42; *t*(78) = 5.16, *p* < 0.001, Cohen’s *d* = 1.18].
