# Supplementary material for: The Differential Impact of Mystery in Nature on Attention: An Oculometric Study
Source: Front Psychol. 2021 Dec 9;12:759616. doi: 10.3389/fpsyg.2021.759616 (PMC8696187; doi:10.3389/fpsyg.2021.759616)
Supplement: Supplementary file 1 [file Table_1.DOCX]

Supplementary Material

*Table S1*

*Mean scores and comparison of the low-level visual properties for both low-mystery and high-mystery images following the method employed by Berman et al. (2014)*

| Low-level visual property | Mystery category | | *t*^a^ | *df* | Cohen’s *d* |
| --- | --- | --- | --- | --- | --- |
|  | Low | High |  |  |  |
| Mean Hue | -0.258 | -0.187 | 0.73 | 78 | 0.17 |
|  | (0.389) | (0.479) |  |  |  |
| Mean Saturation | 0.158 | 0.128 | -3.99*** | 78 | -0.90 |
|  | (0.037) | (0.029) |  |  |  |
| Mean Brightness | 0.565 | 0.532 | -3.96*** | 78 | -0.90 |
|  | (0.033) | (0.041) |  |  |  |
| Hue *SD* | 1.168 | 1.132 | -0.54 | 78 | -0.12 |
|  | (0.240) | (0.335) |  |  |  |
| Saturation *SD* | 0.093 | 0.083 | -2.04* | 78 | -0.46 |
|  | (0.021) | (0.024) |  |  |  |
| Brightness *SD* | 0.207 | 0.193 | -1.72 | 78 | -0.39 |
|  | (0.033) | (0.041) |  |  |  |
| Entropy | 7.500 | 7.376 | -2.30* | 78 | -0.52 |
|  | (0.217) | (0.263) |  |  |  |
| Straight Edges | 0.078 | 0.057 | -2.90* | 78 | -0.66 |
|  | (0.033) | (0.032) |  |  |  |
| Non-Straight Edges | 0.098 | 0.107 | 1.23 | 78 | 0.28 |
|  | (0.031) | (0.031) |  |  |  |
| *Note.* Standard Deviations appear in parentheses below means.  ^a^The test performed to compare the mean scores for each low-level visual property between the two image sets was an independent *t*-test.  * *p* < .05 *** *p* < .001 | | | | | |
